# Bioleaching of lanthanum from nickel metal hydride dry battery using siderophores produced by *Pseudomonas* sp.

**DOI:** 10.1007/s11274-025-04250-9

**Published:** 2025-01-16

**Authors:** Amany S. Hegazy, Hoda M. Soliman, Amr M. Mowafy, Attiya H. Mohamedin

**Affiliations:** 1 Botany Department, Faculty of Science, Mansoura University, Mansoura, 35516 Egypt; 2 Department of Biological Sciences, Faculty of Science, New Mansoura University, New Mansoura City, Egypt

**Keywords:** Bioleaching, Lanthanum, Nickel metal hydride battery, Optimization, *Pseudomonas*, Siderophores.

## Abstract

There is still much to be learned about the properties of siderophores and their applications. This study was designed to characterize and optimize the production of the siderophore produced by a marine bacterium *Pseudomonas* sp. strain ASA235 and then evaluate their use in bioleaching of rare earth elements (REEs) from spent Nickel–metal hydride (NiMH) batteries. The results of both Tetrazolium and Arnowʼs tests indicated that the test organism produces a mixed-type siderophore of pyoverdine family, a result that was confirmed by FT-IR and MALDI-TOFF analyses. Optimization of pH, temperature, incubation period, and iron concentration for siderophore production led to a noticeable shift from 44.5% up to 91% siderophore unit when the test bacterium was incubated at 28 °C and pH 7 after 72 h in the absence of iron. The purified siderophore showed the ability to bleach about 14.8% of lanthanum from the anode of the NiMH battery along with other elements, although in lower amounts. This data put siderophores in distinct focus for further prospective studies intending the bioleaching of such precious elements. The scaling up of this process and optimization would make a big difference in such a green bioleaching strategy, allowing us to recover such precious elements in an environmentally friendly way.

## Introduction

Iron is a necessary element for all life forms. It is essential for many biological activities, including respiration, photosynthesis, nitrogen fixation, DNA metabolism, protein activity, and fatty acid synthesis (Balk and Schaedler 2014; Saha et al. 2016; Tripathi et al. 2018). Iron, as a transition metal, is available as ferrous (Fe II) and ferric (Fe III), which the latter is generally insoluble under aerobic conditions, making it unavailable for organisms (Morrissey and Guerinot 2009; Kanwar et al. 2021).

Microorganisms have developed various strategies to survive under iron limitation conditions. Among these mechanisms is the production of siderophores. The term siderophore is a Greek word describing an iron carrier with low molecular weight multidentate ligand that functioned as Fe(III) chelating agent (Saha et al. 2016; Tripathi et al. 2020). Based on the function groups, siderophores can be divided into three basic categories: hydroxamate, catecholate, and carboxylate. Moreover, a fourth type of siderophore, known as the mixed-type siderophore, comprises more than one iron-binding moiety (Wilson et al. 2016; Sorlin et al. 2024).

Low iron availability triggers the production of siderophores, which are then released outside the cell to chelate iron and facilitate its entry and release within the cell (Sarvepalli and Korrapati 2023). Temperature, pH, and FeCl_3_ concentration may be among the factors that influence the production of siderophores (Saha et al. 2013).

Recently, significant attention has been given to microbial siderophores for their applications across various disciplines, particularly agriculture, such as the potential use of siderophore as a biocontrol agent against phytopathogens (Höfte 2021; Deb and Tatung 2024) and bioleaching in which siderophore produced by *Pseudomonas mendocina* had been used to mobilize iron from a variety of minerals including aluminosilicate clay minerals, asbestos cement, and smectite (Ams et al. 2002; Ferret et al. 2014; Obmiński 2022). Moreover, pyoverdine, a mixed-type siderophore produced by *Pseudomonas* sp, has been used in the bioremediation of metallic trace elements (MTEs) from contaminated soils, and it was also able to chelate with Cd(Π) and reduce Cd accumulation in plants (Mazari et al. 2024; Mei et al. 2024). Compared to traditional hydrometallurgy processes, bioleaching of rare earth elements, particularly from old batteries like Ni-Cd and Lithium batteries, is of great interest. As conventional methods of hydrometallurgy cause serious environmental problems of increasing energy consumption and the use of large amounts of additional chemicals, bio-metallurgy or bioleaching has been considered an alternative emerging green technology for its higher efficiency, low cost, eco-friendly and simplicity (Mowafy 2020). Bio-metallurgy uses microorganisms like bacteria and fungi or their products like siderophores to extract metals in large quantities from their sources (Benzal et al. 2020). Several electric devices are powered by batteries; however, serious environmental concerns are raised about the disposal of expended ones that would release heavy metals, leading to health problems for all living organisms (Bernardes et al. 2004). The use of nickel-metal hydride (NiMH) batteries continues to increase in the global market due to their efficiency and less toxic remains compared to NiCd batteries. The management of the spent NiMH batteries is an essential issue from two sides: the proper disposal and the re-use of the precious components, particularly RREs. Thermal melting and mechanical processing to extract valuable metals from NiMH batteries are expected to yield profits of 2,329 and 2,531 USD per ton (Lin et al. 2016).

By focusing on the properties of siderophores produced by *Pseudomonas* sp. strain ASA235, this study aims to explore their effectiveness in extracting rare earth elements from the NiMH battery components, a target that would not be attained before recognizing the chemical nature and optimizing the conditions for the production. The study seeks to contribute to the development of sustainable and efficient methods for recycling valuable rare earth elements from waste sources like nickel metal hydride batteries, thereby addressing environmental concerns and promoting resource conservation in the context of electronic waste management.

## Materials and methods

Chrome azurol S (CAS), N-(2-hydroxyethyl) piperazine-N’-2-ethane sulfonic acid (HEPES), and hexadecyltrimethylammonium bromide (HDTMA) were all provided by Sigma-Aldrich. All other chemicals used in this study were analytical grade supplied by local companies. Glasswares were rinsed with 6 N HCl before being used to get rid of iron traces. Distilled de-ionized (DI) water was used to prepare all buffers and reagents.

### Microorganisms and growth conditions

The bacterial strain was isolated from a deep-Red Sea core soil sediment sample and molecularly identified as *Pseudomonas* sp. strain ASA235 and submitted to the gene bank (assigned an accession number of MH580294). This strain was selected among others for its ability to produce a significant amount of siderophores in the initial screening (data not shown). The test strain was maintained in 30% glycerol at -20 °C till use.

### Qualitative and quantitative assays for siderophore production

For the qualitative assay, the ability of *Pseudomonas* sp. strain ASA235 to produce siderophore was examined by using CAS-MM9 agar medium containing solution 1(CAS solution/100 ml: 60.5 mg CAS, 0.16 mg FeCl_3_ and 72.9 mg HDTMA) (Chaitanya et al. 2014; Das and Barooah 2018), solution 2 (23.8 g of HEPES, and 20 g agar in 750 ml) Mirabello (2006), and solution 3 ( 150 ml MM9 medium containing 3 g asparagine, 4 g glucose, 10 g NH_4_Cl, 0.4 g NaCl, 0.3 g KH_2_PO_4_, 0.493 g MgCl_2_.6H_2_O, 0.055 g CaCl_2_ and 1 g yeast extract) (Nudel et al. 2001). All solutions were prepared, sterilized individually, and mixed immediately after sterilization (one liter). *Pseudomonas* sp. strain ASA235 was inoculated in CAS-MM9 agar medium and incubated at 37 °C for 24 h. The color change from blue to yellow/orange around the isolate indicated siderophore production.

For quantitative assays, the strain was cultured in MM9 broth medium at 37 °C, 150 rpm for 24 h. The supernatant obtained by centrifugation was mixed with an equal volume of CAS reagent (CAS solution/100 ml: 0.009 g CAS, 0.016 mg FeCl_3,_ 0.017 mg HDTMA, and 5.95 g HEPES) prepared according to Schwyn and Neilands (1987) and Mirabello (2006). The optical density was monitored at 630 nm, and the results were represented as siderophore unit (SU%) according to the following formula:

1 $$\:\text{\%}\:\text{S}\text{i}\text{d}\text{e}\text{r}\text{o}\text{p}\text{h}\text{o}\text{r}\text{e}\text{s}\:\text{u}\text{n}\text{i}\text{t}\:=\:\left[\right(\text{A}\text{r}\hspace{0.17em}-\hspace{0.17em}\text{A}\text{s})/\text{A}\text{r}]\:\times\:\:100$$

Ar is the reference absorbance at 630 nm (CAS reagent with uninoculated MM9 medium), and As is the sample absorbance at the same wavelength (Kejela et al. 2017).

### Detection of siderophore type

The nature of the siderophore was identified using chemical tests. Tetrazolium and Arnowʼs tests were used to discriminate between hydroxamate and catecholate-type siderophores, which is confirmed by the FeCl_3_ test, while Shenker’s test was used to determine carboxylate siderophores. *Pseudomonas* sp. was incubated at 37 °C, 150 rpm for 24 h in MM9 broth medium, and then the following experiments were conducted using the cultural supernatant obtained by centrifugation.

For tetrazolium test, 1 ml of the culture supernatant was combined with 8 mg of tetrazolium salt and 1 drop of 2 N NaOH. The appearance of a deep red color indicates the presence of a hydroxamate siderophore (Snow 1954).

For Arnow’s test, 1 ml of the culture supernatant was mixed with 0.1 ml of 0.5 N HCl, followed by 0.5 ml of ammonium molybdate reagent (containing 10 g NaNO₂ and 10 g Na₂MoO₄·2 H₂O in 50 ml of DI water) and 1 ml of 1 N NaOH. The appearance of pink/red color indicates the presence of catecholate-type siderophores (Arnow 1937).

In FeCl₃ test, 1 ml culture supernatant was combined with 1 ml of 2% ferric chloride solution, and then the spectrum was monitored using Jenway UV/VIS spectrophotometer 7305. The ferric hydroxamate complex produces an orange color with a λ_max_ of 420–450 nm, while the ferric catecholate complex forms a wine color with a λ_max_ of 495 nm (Neilands 1981).

In Shenker’s test, 1 ml culture supernatant was combined with 1 ml of 250 mM CuSO₄ and 2 ml acetate buffer pH 4, and the spectrum was observed using Jenway UV/VIS spectrophotometer. The carboxylate siderophore shows a characteristic peak in the range of 190–280 nm (Shenker et al. 1992).

#### Optimization of growth and siderophore production conditions

In this section, the growth physical factors were optimized for siderophore(s) production. In addition, the iron concentration effect was also monitored.

#### The influence of different initial pH degrees

The influence of different pH degrees was monitored on the growth and siderophore production of *Pseudomonas* sp. grown on MM9 broth medium prepared with three pH degrees: 5, 7, and 8. The inoculated cultures were kept at 37 °C, 150 rpm for 24 h. As previously mentioned, the quantitative assessment of SU was performed using the CAS assay, in which the culture supernatant obtained after centrifugation was mixed with an equal volume of CAS reagent.

#### The effect of temperature and incubation time using the response surface methodology (RSM)

Central Composite Design (CCD) was used to determine the optimum temperature and incubation time providing better bacterial growth for siderophore production. Each variable in the CCD matrix had five levels with five central points and star points to estimate the curvature. Table 1 illustrates the levels of each variable under investigation. A second-order polynomial model was developed to predict the optimum conditions for growth and siderophore unit.

2 $$\:\text{Y}\hspace{0.17em}=\hspace{0.17em}0\hspace{0.17em}+\hspace{0.17em}\:\text{i}\:\text{X}\text{i}\hspace{0.17em}+\hspace{0.17em}\:\text{i}\text{i}\:\text{X}\text{i}\text{i}\hspace{0.17em}+\hspace{0.17em}\:\text{i}\text{j}\text{X}\text{i}\text{j}$$

Y is the response, βi are the regression coefficients for each factor, βii are the regression coefficients for square effects, and βij are the regression coefficients for interaction.

CCD design generated 13 experiments with five replicates of central points. This experiment was conducted by inoculating the test strain in 50 ml of MM9 broth medium at pH 7 and was incubated under various combinations of temperature and incubation time, as described in Table 2. Analysis of variance (ANOVA) was carried out using the Design Expert 12 statistical package (Stat Ease, Inc., Minneapolis, MN, USA). Based on the analysis of the results, a validation experiment for the CCD was performed.

**Table 1:** Variables and their levels used for CCD experiment for temperature and incubation time

Variables	Symbol	Levels				
		-2	-1	0	+ 1	+ 2
Temperature (°C)	X1	26	28	32.5	37	38.8
Incubation time (h)	X2	14	24	48	72	82

#### The effect of iron on siderophore production and bacterial growth

The MM9 broth medium used in this experiment was supplied with different FeCl_3_ concentrations (0, 25, 50, 75, and 100 µM). The inoculated cultures were incubated under the verified optimum conditions, as indicated in the results Sect. (28 °C for 72 h).

### Siderophores purification and chemical characterization

For purification of siderophores, MM9 medium was prepared, inoculated, and incubated at 28 °C, 150 rpm for 72 h without iron chloride addition (the verified optimum production conditions as will be shown in the result section). After centrifugation at 6000 rpm for 30 min, the recovered supernatant pH was shifted to 2 using 6 M HCl to decrease the solubility of siderophores. Amberlite XAD-2 column (3 × 50 cm) was used for purification after preparation. First, the column matrix was soaked in DI water for 24 h at room temperature. Then, equilibration was attained with four-bed volumes of DI water. After loading the acidified supernatant, the column was washed with three-bed volumes of DI water. Elution was attained by passing approximately 500 ml of 50% methanol. Altogether, ten fractions of 50 ml were collected (Srivastava et al. 2022). The flow-through, DI water wash, and all fractions were collected and tested for siderophore content using the previously described CAS assay. The fractions that showed higher values of SU% were combined and then dried on a freeze drier (Freezone, LABCONCO, USA) and kept at -20 °C for future work.

## FT-IR analysis

The purified siderophore sample was analyzed using ATR-FTIR (Attenuated Total Reflectance Fourier-Transform Infrared). FT-IR spectrum was obtained using Bruker FT-IR spectrophotometer (INVENIO S, Germany). The resolution of the spectra was 4 cm^− 1^, and the wavenumber range of 400–4000 cm^− 1^ was applied during the analysis.

### Matrix-assisted laser desorption/ionization time-of-flight mass spectrometry (MALDI-TOF MS) analysis

The purified siderophore sample was mixed with α -cyano-4-hydroxycinnamic acid in 50% acetonitrile/0.1%TFA (trifluoroacetic acid) (1:1) and spotted on 100-well stainless steel MALDI plate and analyzed in a positive mode. MALDI-MS analysis was performed using a MALDI-TOF Scientific Analysis Instruments (SAI- LT2).

### Bioleaching of metals with siderophores from spent batteries

#### Spent batteries powder preparation

The spent nickel–metal hydride (NiMH) batteries were manually dismantled, dried in the air, ground, and sieved to obtain a mesh size of less than 100 μm.

## Bleaching experiment

About 0.1 g of the purified siderophore was dissolved in 10 ml DI water and mixed with 0.1 g of NiMH battery powder. The incubation took place in a shaker incubator at 28 °C and 150 rpm for 8 days. The mixture was then centrifuged at 4000 rpm for 10 min, and the leftover battery powder was obtained, washed with DI water and dried in an oven at 80 °C. As a control, 0.1 g of battery powder was mixed with 10 ml of DI water without siderophores and subjected to the same conditions described. The dried leftover powder of the battery sample subjected to both conditions was digested using a solution of 5 ml of concentrated nitric acid and 5 ml of DI water. The mixture was left overnight at room temperature. Then, in a fume hood, the mixture was digested. After cooling, the solutions were diluted with DI water and filtered to eliminate the insoluble residues. The REEs in the filtrates were measured using inductively coupled plasma atomic emission spectroscopy (ICP-AES) to determine the amounts of metals.

The removal efficiency percentage (E%) was calculated as follows:

3 $$\:\text{R}\text{e}\text{m}\text{o}\text{v}\text{a}\text{l}\:\text{e}\text{f}\text{f}\text{i}\text{c}\text{i}\text{e}\text{n}\text{c}\text{y}\:\left(\text{E}\text{\%}\right)\:=\:\left[\right(\text{C}\text{o}\:\--\:\text{C}\text{f})/\text{C}\text{o}]\:\times\:\:100$$

Co and Cf are the concentrations of the metal before and after siderophore treatment, respectively.

### Statistical analysis

The mean and standard error were calculated from triplicate experiments. One-way ANOVA was conducted to analyze the results obtained from pH and FeCl₃ concentration optimization experiments to identify differences between treatments at a significance level of *p* ≤ 0.05 using CoStat software (798 Lighthouse Ave. PMB 329, Monterey, CA, 93940, USA) and Duncan’s test. Similarly, one-way ANOVA was applied for the results of the temperature and incubation time optimization experiment at *p* ≤ 0.05 using Design Expert software (Stat Ease, Inc., Minneapolis, MN, USA) and the CCD method.

## Results

**Fig. 1:**
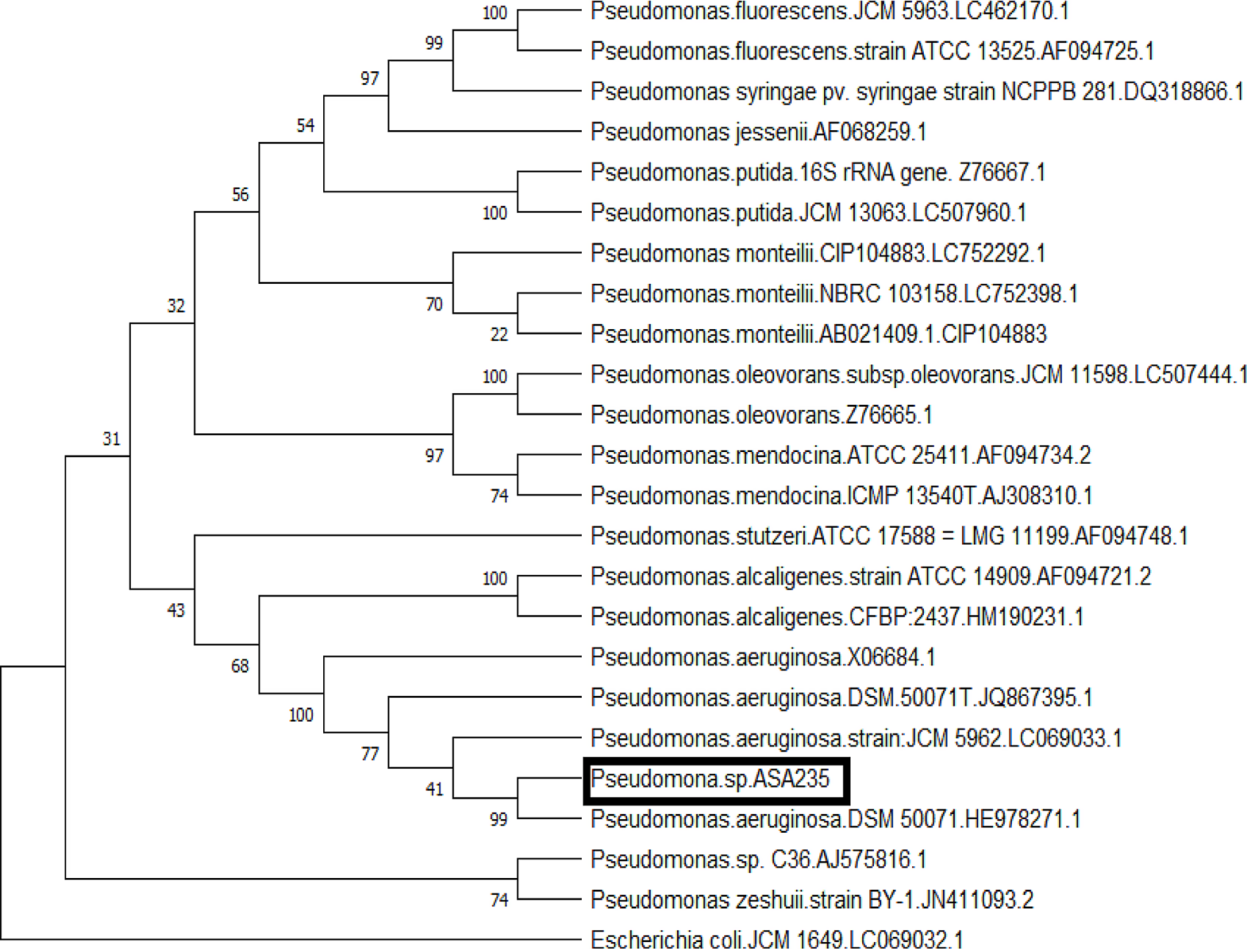
Phylogenetic tree of the bacterial strain used in this study based on 16S rRNA sequence displaying its position to the closely related species of the genus *Pseudomonas*. The tree was constructed using MEGA X software based on the maximum likelihood method. *E. coli*.JCM_1649_LC069032.1 was used as an external reference

The bacterial strain used in our study was molecularly identified by 16 S rRNA sequence analysis. The results show that the strain has a high sequence identity of 99% to *Pseudomonas aeruginosa* DSM_50071T_HE978271.1 type strain. (Fig. 1). After being submitted to GenBank, the obtained sequence was assigned the accession number MH580294 in the GenBank.

### Siderophore production

**Fig. 2:**
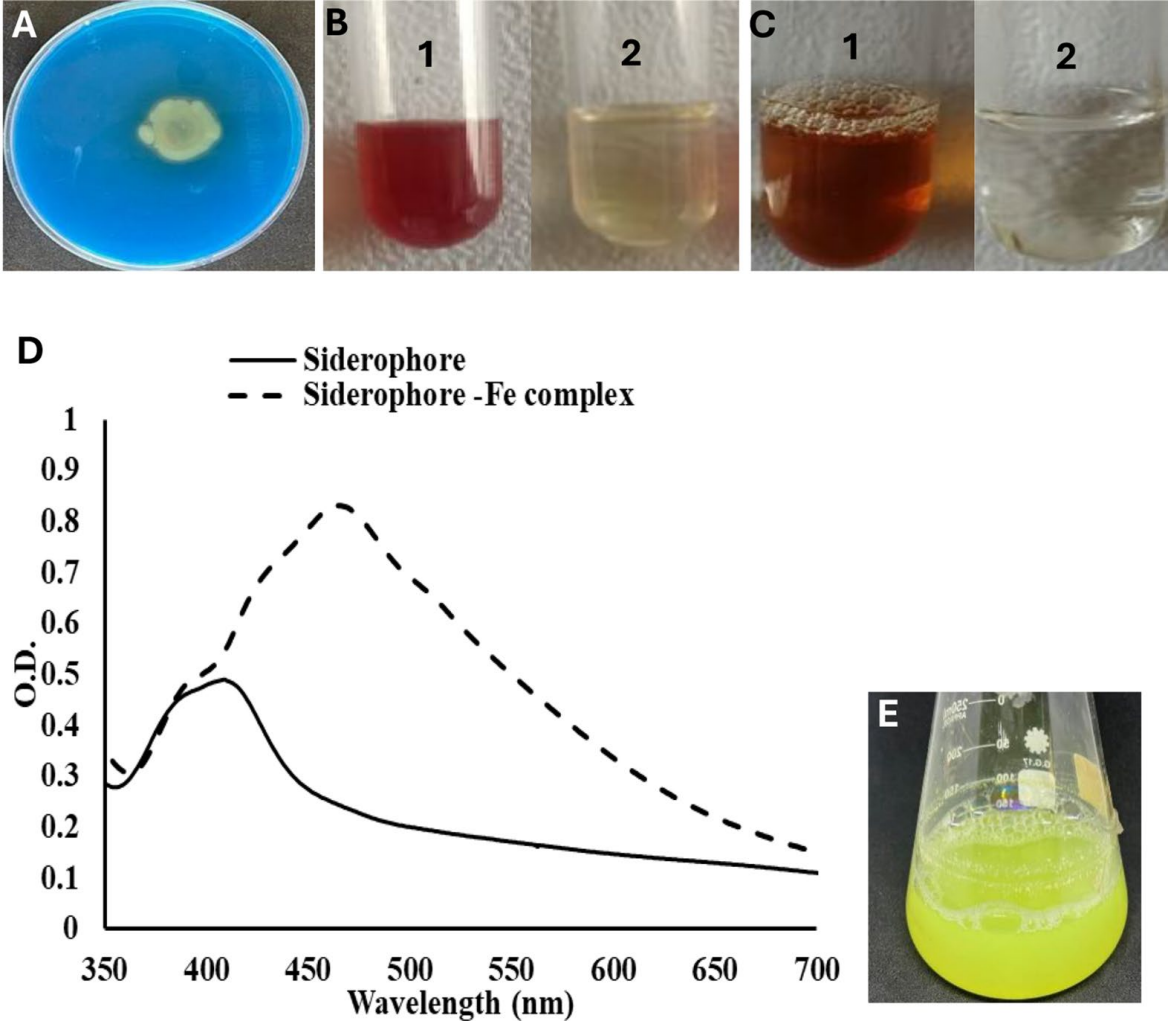
**(A)** CAS agar assay showing the production of siderophores by *Pseudomonas* sp. and the change of the blue color of CAS-agar medium to the yellow color. (**B1)** The formation of the deep red color of the bacterial siderophore indicates a positive tetrazolium test, while (**B2)** is a negative control showed no color change. (**C1)** The formation of the deep yellow-brown color of the bacterial siderophore indicating a positive Arnow´s test, while (**C2)** is a negative control. (**D)** UV–VIS spectrum of siderophore alone (connected line) and siderophore-iron complex (dashed line) showing the characteristic maximum absorbance at 410 nm and 468 nm, respectively. **E)** The developed siderophore with lemon green pigment

The results shown in Fig. 2.A demonstrated the test strain’s ability to produce siderophores as indicated by yellow halo development around the colony when inoculated in CAS agar medium. The color change indicates iron chelation from CAS dye. This strain was able to produce 44.5% siderophore units according to the data obtained from CAS quantitative assay. This value will be further optimized.

### Detection of siderophore function groups

The experiments performed to explore the chemical nature of siderophore showed positive results for both tetrazolium and Arnowʼs tests with a characteristic red color and deep yellow-brown color, respectively, as shown in Fig. 2B and C, in addition to a characteristic maximum absorbance at 468 nm in the FeCl_3_ test (Fig. 2D– dashed line). The λ_max_ obtained at 468 nm, neither 420–450 for hydroxamate nor 495 nm for catecholate, indicated that the tested siderophore is a mixed type “containing hydroxamate and catecholate group”. The results obtained in Arnow´s test might be due to the substitution of vicinal diols in positions 3 and 4, leading to the formation of an unstable yellow compound as a final product of this reaction (Ferreira et al. 2018). The λ_max_ detected at 410 nm for the siderophore alone (Fig. 2D– connected line) indicated that this siderophore belongs to the pyoverdine group.

### Optimization of siderophore production

**Fig. 3:**
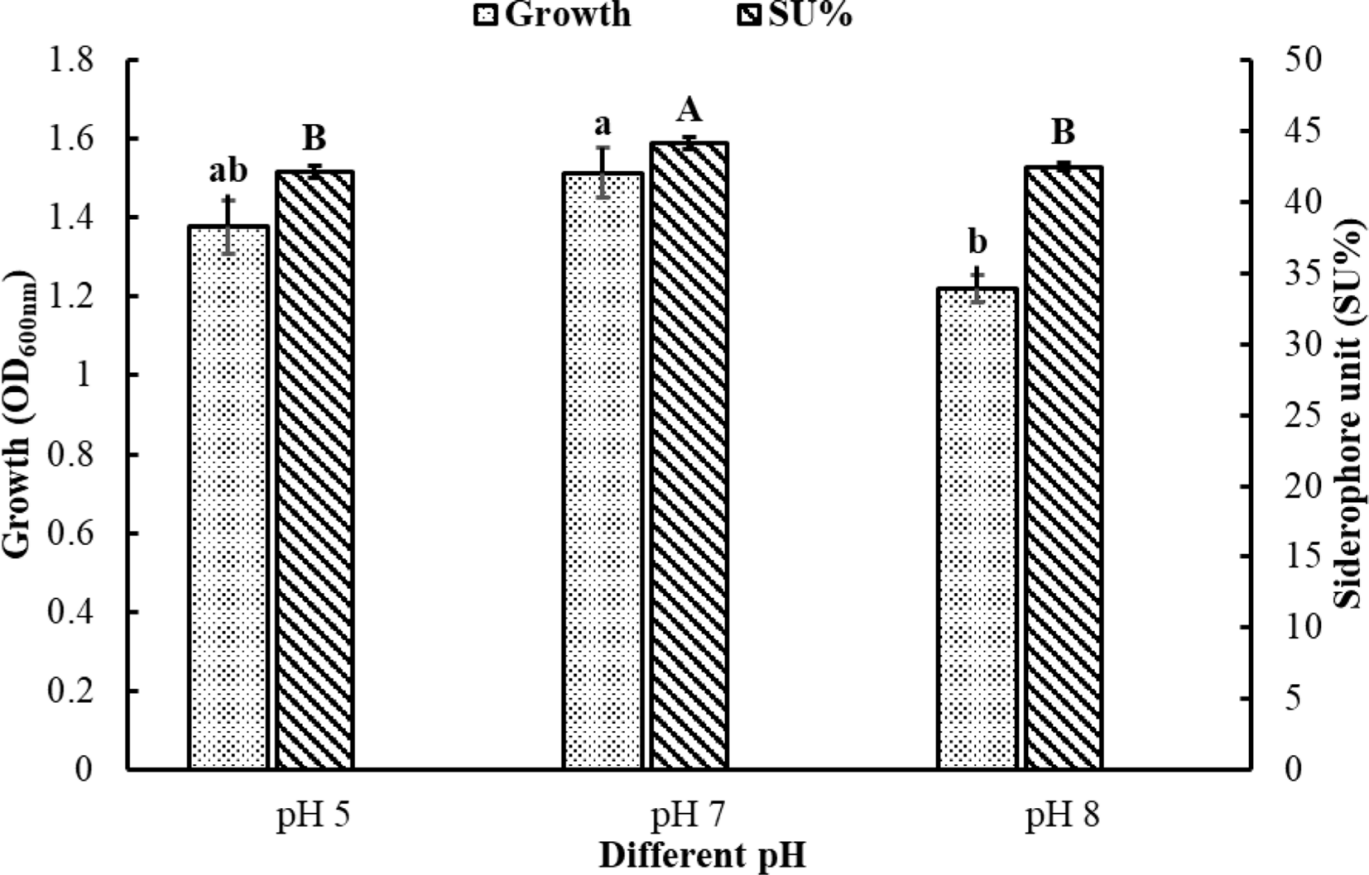
Effect of different pH degrees on the growth of *Pseudomonas* sp. and its productivity of siderophore. The results were expressed as mean of triplicates ± standard error (S.E). According to Duncan’s test, different letters in small and capital styles indicate significant differences in growth and SU%, respectively, in response to different pH values (*p-*value ≤ 0.05)

The data represented in Fig. 3 revealed that the highest siderophore production (44.5% SU) was obtained at pH 7.0, which was significantly different from those obtained at pH 5 and 8 (42.1% and 42.4%) (*p* < 0.05), whereas the highest growth expressed by highest OD (1.5) was obtained at pH 7.0, compared to that obtained at pH 8 (1.22) (*p* < 0.05).

A CCD matrix was applied to test the effect of temperature and incubation time on siderophore production (SU%) and bacterial growth “in terms of OD_600nm_” (Table 2).

**Table 2:** Matrix and responses of the CCD experiment to show the effect of temperature and incubation time on siderophore unit production and growth of *Pseudomonas* Sp.

Runs	Variables in coded and uncoded values				Responses	
	**Temperature** (°C)		**Incubation time** (h)		**Growth** (OD_600nm_)	**Siderophore unit** (SU%)
	Coded values	Uncoded values	Coded values	Uncoded values		
1	-1	28	-1	24	1.872	78.09%
2	+ 1	37	-1	24	1.016	44.5%
3	-1	28	+ 1	72	3.216	91.07%
4	+ 1	37	+ 1	72	2.024	85.27%
5	-2	26	0	48	3.100	75.39%
6	+ 2	38.8	0	48	1.393	63.4%
7	0	32.5	-2	14	0.953	81.93%
8	0	32.5	+ 2	82	2.500	91.33%
9	0	32.5	0	48	1.878	84.56%
10	0	32.5	0	48	1.880	84.03%
11	0	32.5	0	48	1.770	82.71%
12	0	32.5	0	48	1.750	82.5%
13	0	32.5	0	48	1.800	83.56%

Trials 3 and 8 showed the maximum siderophore production, while the highest growth was achieved in trials 3 and 5. ANOVA of the linear, quadratic effects and the interaction among factors are shown in Table 3. Factors with a *p*-value less than 0.05 were considered significant.

**Table 3:** ANOVA table for the CCD experiment showing the effect of temperature and incubation time on siderophore unit production and growth of *Pseudomonas* sp.; statistical terms with *p*-values ≤ 0.05 were displayed in **bold**

Variable	Responses					
	Growth (OD_600nm_)			Siderophore unit (SU%)		
Source	Sum of squares	F value	*p*-value	Sum of squares	F value	*p-*value
Model	5.48	197.04	**< 0.0001**	1603.92	7.69	**0.0092**
A-Temp (°C)	2.49	447.52	**< 0.0001**	396.86	9.51	**0.0177**
B-Incubation Time (h)	2.58	463.24	**< 0.0001**	561.86	13.47	**0.0080**
AB	0.0282	5.08	0.0590	193.07	4.63	0.0685
A^2^	0.3579	64.36	**< 0.0001**	429.61	10.30	**0.0149**
B^2^	0.0077	1.38	0.2790	4.01	0.0961	0.7656

The CCD design *P-*value of 0.0092 and < 0.0001 means that the model is significant for both responses. It detects the most significant factors that affect siderophore production and bacterial growth at a level of confidence of 95%. The second-order polynomial models that predicted the siderophore production and the microbial growth as a function of the experimental variable are shown in Eqs. (4 & 5)

4 $$\:\text{S}\text{i}\text{d}\text{e}\text{r}\text{o}\text{p}\text{h}\text{o}\text{r}\text{e}\:\text{p}\text{r}\text{o}\text{d}\text{u}\text{c}\text{t}\text{i}\text{o}\text{n}\hspace{0.17em}=\hspace{0.17em}83.47-7.04\text{*}\text{A}\hspace{0.17em}+\hspace{0.17em}8.38\text{*}\text{B}\hspace{0.17em}+\hspace{0.17em}6.95\text{*}\text{A}\text{B}-\:7.86\text{*}\text{A}^{2}\:+0.7590\text{*}\text{B}^{2}$$

5 $$\:\text{G}\text{r}\text{o}\text{w}\text{t}\text{h}\hspace{0.17em}=\hspace{0.17em}1.82\hspace{0.17em}-\hspace{0.17em}0.557\text{*}\text{A}\hspace{0.17em}+\hspace{0.17em}0.567\text{*}\text{B}\:-\hspace{0.17em}0.084\text{*}\text{A}\text{B}\hspace{0.17em}+\hspace{0.17em}0.227\text{*}\text{A}^{2}\:-\hspace{0.17em}0.033\text{*}\text{B}^{2}$$

According to ANOVA analysis (Table 3), the main effect of temperature and incubation time were the most significant factors affecting siderophore production, with *p-*values (0.0177 and 0.0080), respectively. The quadratic effect of temperature was significant, and the *p-*value was 0.0149.

From ANOVA analysis (Table 3), the main effect of temperature and incubation time was found to be the most significant factor affecting bacterial growth, with a *p-*value (< 0.0001). The quadratic effect of temperature was significant, and the *p***-**value was < 0.0001.

**Fig. 4:**
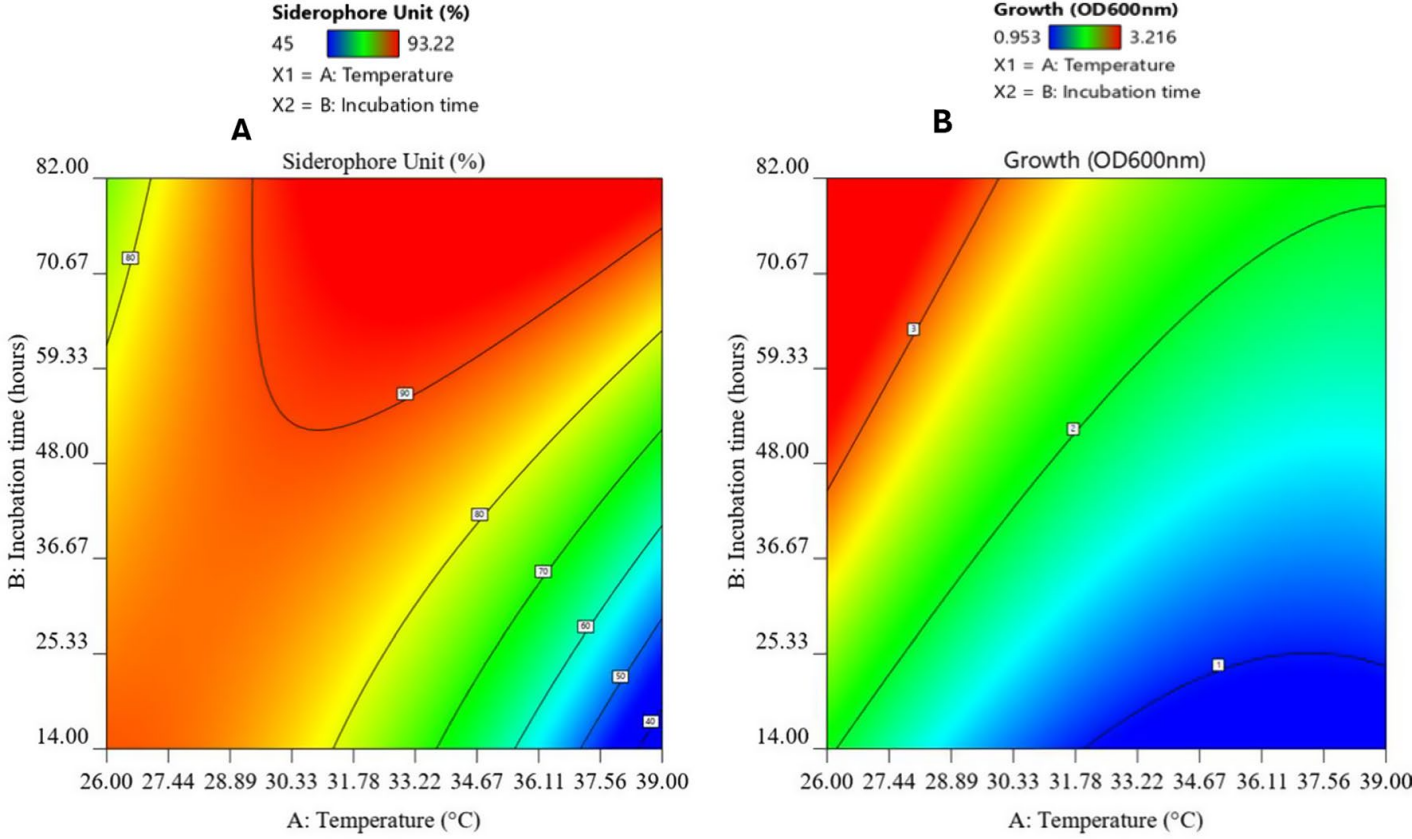
Color contour plot showing the interaction between the tested variables (temperature and incubation time) for optimum responses of (**A**) siderophore production and (**B**) bacterial growth

The interaction between the examined factors on the siderophore production detected as SU% and the bacterial growth detected as (O.D._600nm_) was created by contour plot (Fig. 4). The deep red color indicates the optimum condition for each response. In terms of the interaction between temperature and incubation time, the optimum temperature range for the calculated response (SU%) was 32.5 °C, while the optimum incubation time was 60–82 h. Regarding the interaction between temperature and incubation time, the optimum temperature range for the calculated response (O.D._600nm_) was 26 °C, while the optimum incubation time was 48–82 h.

The optimum conditions for each response variable were estimated individually using the generated mathematical models and the interaction 3D surface response graphs. The model predicted that maximum siderophore production and optical density could be achieved by using different combinations of different temperatures and degrees of incubation time. The prediction was verified experimentally, and the software predicted that the maximum siderophore production was achieved when the temperature was 34 °C and the incubation time was 82 h. The model overestimated the SU% since the actual percent attained (90%) was less than the predicted value of 96.89% of the SU%. The ideal temperature and incubation period for bacterial growth and siderophore production were 28 °C and 72 h.

### The influence of iron concentration on the siderophore production and bacterial growth

**Fig. 5:**
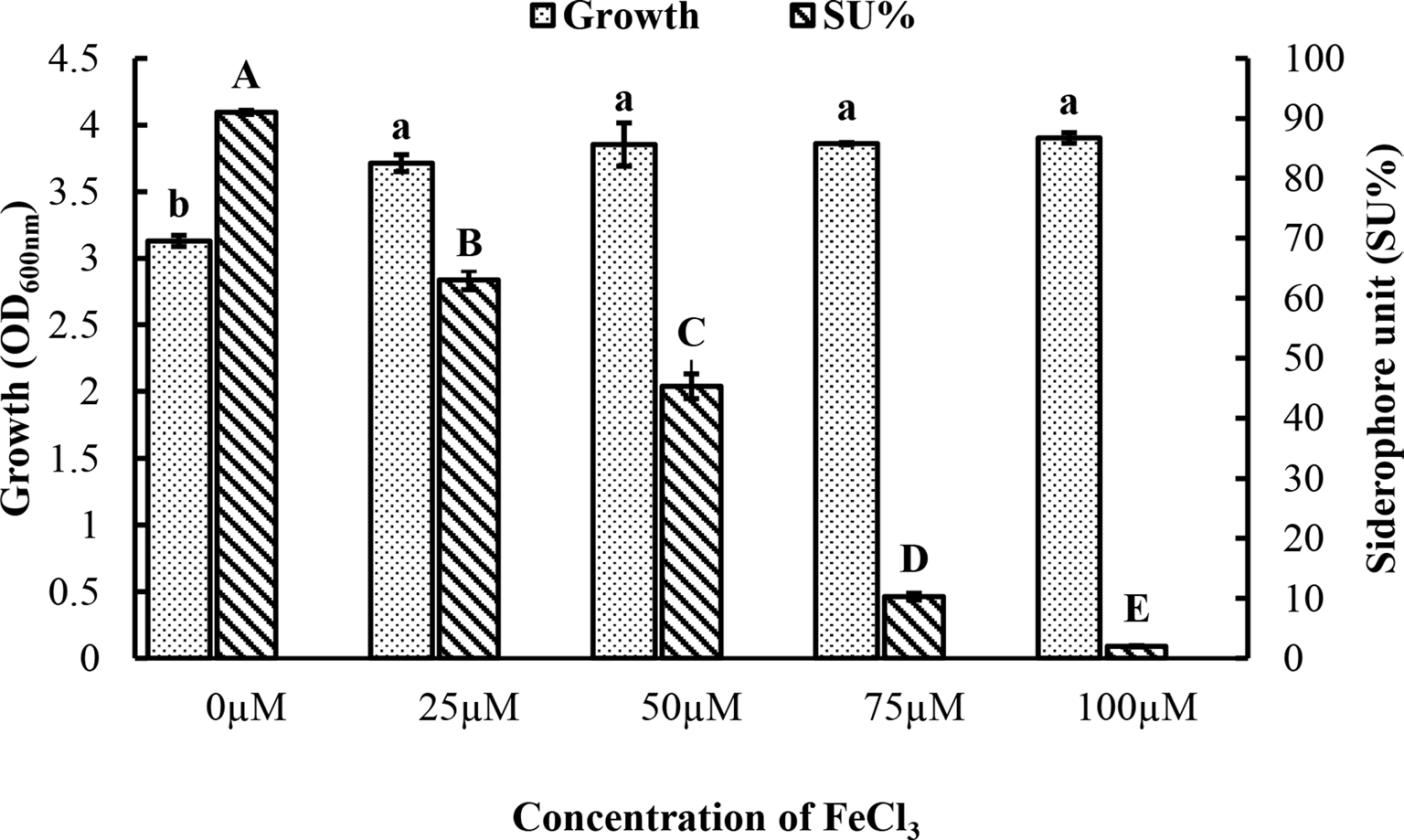
Influence of different concentrations of FeCl_3_ on the production of siderophore and growth of *Pseudomonas* sp. The results were expressed as mean of triplicates ± standard error (S.E). Duncan’s test revealed that different letters differ significantly from each bar (*p-*value ≤ 0.05). Different letters in small and capital styles indicate the significant differences between different values of growth and SU%, respectively, according to Duncan’s test

Siderophore is known to be produced in response to low iron concentrations. Figure 5 shows a significant increase in bacterial growth at 25, 50, 75, and 100 µM compared to control (zero µM). There was a slight increase by increasing the concentration of FeCl_3_ as the minimum growth (OD_600nm_) was 3.13 at the control, and the maximum was 3.9 at 100 µM.

On the contrary, there is a significant decrease in siderophore production at 25, 50, 75, and 100µM compared to control. There was a decrease by increasing the concentration of FeCl_3_ as the minimum SU% was 2% at 100µM and the maximum SU% was 91% at control.

### Siderophores purification

After column purification, CAS assay was used to determine whether siderophores were present in these fractions. Only five fractions out of ten exhibited yellow coloration, and the CAS test revealed that siderophore was present. These fractions were mixed, dried on a freeze drier, and kept for further tests.

### FT-IR analysis of purified siderophore

**Fig. 6:**
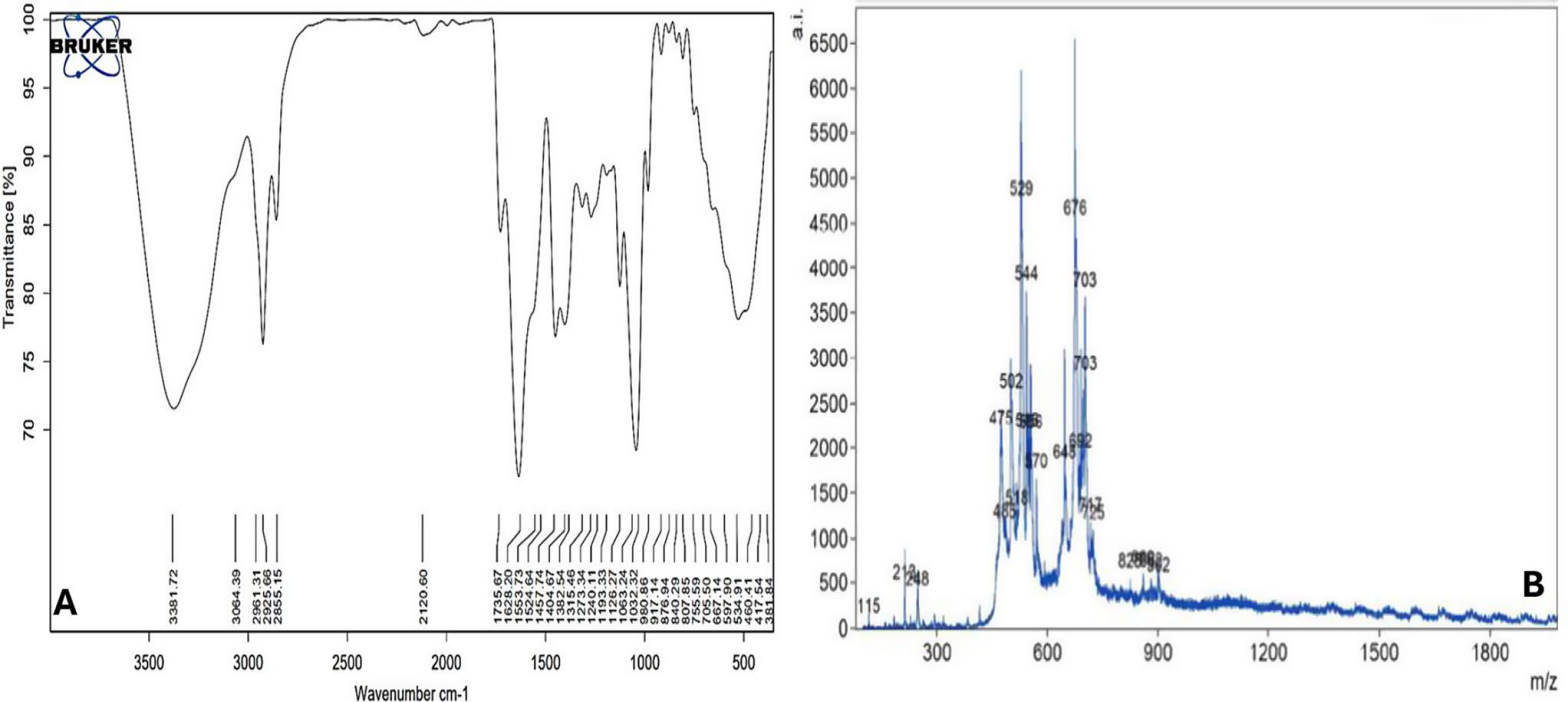
Chemical characterization of the purified siderophore where (**A**) FT-IR spectrum of the purified siderophore and (**B**) MALDI-TOF MS analysis of the purified siderophore

The FTIR spectrum of the purified siderophore (Fig. 6. A) revealed that the broad absorption band at 3381 cm^− 1^ indicated the presence of hydroxyl groups (O-H), likely associated with alcohols, phenols, or carboxylic acids involved in hydrogen bonding. The 3064 cm^− 1^ and 2961–2855 cm^− 1^ peaks confirmed aromatic and aliphatic C-H stretching vibrations, respectively. A strong carbonyl peak at 1735 cm^− 1^ suggested the presence of carbonyl groups (C = O), commonly found in aldehydes, ketones, esters, or carboxylic acids. The band at 1628 cm^− 1^ is characteristic of amide carbonyl groups, suggesting the presence of peptide bonds. Additionally, the peaks around 1553 and 1524 cm^− 1^ indicate aromatic C = C stretching vibrations, consistent with the aromatic nature of pyoverdines.

The FTIR analysis results agree with the known structural features of pyoverdines. The presence of hydroxyl groups, carbonyl groups, and aromatic rings is consistent with pyoverdines’ peptide backbone and chromophore moiety. Identifying amide carbonyl groups further supports the peptide nature of these molecules. Moreover, the hydroxyl group is likely associated with phenols, and the amide carbonyl group confirms the presence of a mixed type siderophore.

### Matrix-assisted laser desorption/ionization time-of-flight mass spectrometry (MALDI-TOF MS) analysis

The mass spectrum of the purified sample obtained by MALDI-TOF MS analysis is shown in Fig. 6. B. Generally doubled charged ions [M + 2 H]^2+^ are detected in the positive ion mass spectra. The purified siderophore comprises two major peaks at m/z 529 and m/z 676 corresponding to the type [M + 2 H]^2+^ ions. Other signals of minor abundance are at m/z 475, 502, 544, 648, and 703.

### Bioleaching of metals with siderophores from spent batteries

The removal efficiency percent (E%) of certain rare earth elements (REEs) in spent NiMH batteries that were bioleached by purified pyoverdine is displayed in Table 4. Lanthanum was the element that recorded the highest removal efficiency percent (14.803%), followed by neodymium (3.922%) and praseodymium (1.105%).

**Table 4:** The removal efficiency percentage of La, Ce, Pr, and nd from spent NiMH battery for the purified pyoverdine extracted from *Pseudomonas* Sp

Treatment	Removal Efficiency Percent (%)			
	La	Ce	Pr	Nd
**Pure Pyoverdine**	14.803	-	1.105	3.922

## Discussion

Integrating the versatile properties of siderophores into the extraction process offers a novel approach to effectively and sustainably bioleaching rare earth elements from intricate substrates, a focal point examined in this study. The marine bacterium identified as *Pseudomonas* sp. strain ASA235 was used to achieve the aim of this study, which was to produce a regarded amount of siderophores (44.5% SU) in the initial screening.

The siderophore produced by *Pseudomonas* sp. was characterized as a mixed-type siderophore, a conclusion obtained by tetrazolium and Arnow’s tests positive results along with FT-IR. *Pseudomonas veronii* was reported to produce the same type of siderophore (Ferreira et al. 2018). The obtained siderophore is characterized by a green–yellow color (Fig. 2E) with a maximum absorbance peak at 410 nm (Fig. 2D), suggesting it belongs to the pyoverdine family. Several *Pseudomonas* strains were reported to produce pyoverdine (Moll et al. 2008; Tank et al. 2012). Another supporting evidence has come from MALDI-TOF MS analysis in which a major peak at m/z 676 [M + H] ^+^ was observed (Fig. 6B). The same m/z value was reported for pyoverdine (Fuchs et al. 2001; Uría Fernández et al. 2003).

In this study, MM9 medium was used for siderophore production optimization experiments because, in several studies, it was nominated as the most suitable culture medium (among six tested medium) to allow siderophore production by *P. putida* due to the compositional similarity to that of the marine environment (Murugappan et al. 2012). Besides Fe concentration, the effect of pH, temperature, and incubation time on productivity have been studied.

The highest siderophore production (44.5% unit) was obtained when incubation started at pH 7. The same finding was reported previously for various *Pseudomonas* strains (Pattan et al. 2017; Monali et al. 2018; Kotb et al. 2024). It is interesting to mention that the final pH measured in all cultures of our study was 7.2–7.6 (regardless of whether the start was pH 5, 7, or 8), and this may be linked in part to asparagine that works as a weak acid or base based on the medium pH (Darnal et al. 2023). Any shift from the neutral pH influences both the bioavailability of iron and bacterial growth, making it the optimum condition for siderophore production (Kumar et al. 2017; Piskin et al. 2022). The changes in pH are linked to the metabolism of carbon and nitrogen sources, which impacts the stability of the siderophore produced by *Pseudomonas aeruginosa*, as mentioned by de Villegas et al. (2002). On the contrary, Murugappan et al. (2012) and Sinha et al. (2019) reported that the optimum production of siderophore by *P. putida* and other bacteria was attained at alkaline pH.

The statistical design used to screen conditions suitable for both siderophore production and bacterial growth revealed that the best conditions for siderophore production were 32.5 °C after 82 h. However, the study was conducted at 28 °C and 72 h, as the difference in siderophore production between the two conditions was minimal, and the chosen conditions supported higher bacterial growth in lesser incubation time. The same result was obtained by *P. aeruginosa* azar11, which showed a progressive increase in siderophore secretion till 72 h to 96 h, which decreased after 120 h (Colombowala and Aruna 2018). *Bacillus subtilis* DR2, *Jeotgalicoccus huakuii*, and *Bacillus amyloliquefaciens* showed the ability to produce the maximum amount of siderophores within 72 h (Ustiatik et al. 2021; Kumari et al. 2022). The 72-hour maximum production of siderophores of test strain in this study might be attributed to the deficiency in iron concentration tuned by washing with HCl to get rid of all iron traces. The variations represented for different strains might be due to their different requirements, growth rates, and responses.

It is interesting to mention that the optimum growth temperature for the test strain was 26–28 °C (Pietikäinen et al. 2005), a temperature that is quite lower than the one found to give optimum siderophore productivity and growth. Generally, when incubated at a temperature closer to its nature, the microorganism shows maximum growth and siderophore secretion (Soares 2022). This might explain the ability of the test strain in this study to yield high siderophores, close to the optimum one when incubated at 28 °C for 72 h. The optimum temperature for siderophore secretion and growth of *Pseudomonas aeruginosa* was reported at 27 °C by El-Sheikh et al. (2011). Meanwhile, Chiadò et al. (2013) reported that the temperature degree (20–25 °C) that was optimum for the growth of *P*. *fluorescens* was higher than the temperature (15–20 °C) that was optimum for pyoverdine production.

The data in Fig. 5 depicts that increasing initial iron concentration positively affects bacterial growth, as the maximum growth was obtained at 100 µM. However, siderophore produced by *Pseudomonas* sp. was significantly repressed as initial Fe (III) concentration increased, with almost no siderophore produced when FeCl_3_ concentration reached 100 µM. The result was similar to that of Yu et al. (2017), indicating almost no siderophore production when *Bacillus* sp. PZ-1 was cultured in a medium containing 100 µM iron. Since siderophores are secreted in response to iron scarcely, iron concentration is considered the most critical and vital factor determining the level of siderophore production (Sulochana et al. 2014). Many studies summarize that siderophore production decreased by increasing iron concentration (Braun and Hantke 2013; Sah et al. 2015; Niehus et al. 2017; Khan et al. 2018; Rajapitamahuni et al. 2023). de Villegas et al. (2002) stated that increasing the concentration of iron had a negative effect on siderophore secretion, especially above 10 µM; however, above this concentration, *Pseudomonas aeruginosa* PSS growth reached its maximal value. Generally, adequate levels of cytoplasmic iron cause promotors of siderophore-related genes to be downregulated, and bacteria can sense iron levels, enabling siderophore inhibition until iron levels become a growth-limiting factor (Sánchez-Jiménez et al. 2023). Studies conducted on different bacterial strains revealed that the range of iron that allows the production of siderophores, as well as the amount of siderophores produced and the concentration that inhibits the productivity, are all species-specific due to different microbial physiological and growth conditions (Rachid and Ahmed 2005; Sayyed et al. 2005; El-Sheikh et al. 2011; Tailor and Joshi 2012).

The purified mixed-type pyoverdine obtained in this study could bioleach lanthanum with 14.80%. Some other elements were leached in minor ratios, such as neodymium (3.92%) and praseodymium (1.10%). To our knowledge, the current report might be the first one indicating the ability of siderophores to leach lanthanum from nickel-metal hydride batteries (NiMHBs), which typically contain lanthanum, cerium, neodymium, and praseodymium (Salehi et al. 2023) along with nickel, iron, and copper representing more than 60% of the battery weight (Lin et al. 2016). This type of battery is widely used even in electric vehicles (Ovshinsky et al. 1993). Like iron, the positive charge of this element makes it able to chemically interact with hydroxamate and catecholate groups in the tested pyoverdine as a mixed-type siderophore (Schijf et al. 2015; Hofmann et al. 2020). Lanthanum’s affinity to siderophore, which means its ability to make a coordination complex, might be the reason behind its leaching in the highest percentage (Andres et al. 1991; Carter et al. 2020). The data represented here might need further fine-tuning based on the relative amount of added siderophore and the entire cell in whole-cell assays. This process would reduce the cost of siderophore purification. The results of this experiment represent evidence that siderophores are a strong candidate for acting as REE chelators. A tri-hydroxamate siderophore produced from *Aspergillus niger* was documented to bioleach lanthanum with 51%, cerium with 50.1%, and other REE from phosphorites (Osman et al. 2019). There is a growing interest in using siderophores in such bioleaching processes (Osman et al. 2019; Mowafy 2020; Vishwakarma and Hait 2024). Several reports indicated the ability of further metabolites such as polysaccharides and protein substances (Naseri and Mousavi 2022) as well as organic acids like oxalic acid, gluconic acid, and citric acid (Ma et al. 2023; Zhou et al. 2024) in the bioleaching of REEs. However, the use of pyoverdine in the purified status put siderophores in distinct focus for further prospective studies intending the bioleaching of such precious elements.

In conclusion, the findings in this study demonstrate the promising capability of the purified mixed-type siderophore produced by *Pseudomonas* sp. strain ASA235 to extract lanthanum and other REEs in few amounts from the spent Nickel-metal hydride batteries. This approach not only showcases the potential for sustainable recovery of valuable resources but also highlights the role of biologically derived compounds in enhancing the efficiency of precious metal extraction processes aiming to reduce or replace, even in part, the harsh physical and chemical treatments. Further exploration and optimization of this method could pave the way for eco-friendly and economically viable strategies in recycling REEs from electronic waste, contributing to a more sustainable future for resource management and conservation.

## Data Availability

No datasets were generated or analysed during the current study.
